# Validation of the Zebrafish Pentylenetetrazol Seizure Model: Locomotor versus Electrographic Responses to Antiepileptic Drugs

**DOI:** 10.1371/journal.pone.0054166

**Published:** 2013-01-14

**Authors:** Tatiana Afrikanova, Ann-Sophie K. Serruys, Olivia E. M. Buenafe, Ralph Clinckers, Ilse Smolders, Peter A. M. de Witte, Alexander D. Crawford, Camila V. Esguerra

**Affiliations:** 1 Laboratory for Molecular Biodiscovery, Department of Pharmaceutical and Pharmacological Sciences, University of Leuven, Leuven, Belgium; 2 Center for Neurosciences, Department of Pharmaceutical Chemistry and Drug Analysis, Vrije Universiteit Brussel, Brussels, Belgium; National Institutes of Health/NICHD, United States of America

## Abstract

Zebrafish have recently emerged as an attractive *in vivo* model for epilepsy. Seven-day-old zebrafish larvae exposed to the GABA_A_ antagonist pentylenetetrazol (PTZ) exhibit increased locomotor activity, seizure-like behavior, and epileptiform electrographic activity. A previous study showed that 12 out of 13 antiepileptic drugs (AEDs) suppressed PTZ-mediated increases in larval movement, indicating the potential utility of zebrafish as a high-throughput *in vivo* model for AED discovery. However, a question remained as to whether an AED-induced decrease in locomotion is truly indicative of anticonvulsant activity, as some drugs may impair larval movement through other mechanisms such as general toxicity or sedation. We therefore carried out a study in PTZ-treated zebrafish larvae, to directly compare the ability of AEDs to inhibit seizure-like behavioral manifestations with their capacity to suppress epileptiform electrographic activity. We re-tested the 13 AEDs of which 12 were previously reported to inhibit convulsions in the larval movement tracking assay, administering concentrations that did not, on their own, impair locomotion. In parallel, we carried out open-field recordings on larval brains after treatment with each AED. For the majority of AEDs we obtained the same response in both the behavioral and electrographic assays. Overall our data correlate well with those reported in the literature for acute rodent PTZ tests, indicating that the larval zebrafish brain is more discriminatory than previously thought in its response to AEDs with different modes of action. Our results underscore the validity of using the zebrafish larval locomotor assay as a rapid first-pass screening tool in assessing the anticonvulsant and/or proconvulsant activity of compounds, but also highlight the importance of performing adequate validation when using in vivo models.

## Introduction

Epilepsy is a common neurological disorder marked by episodic seizures as a result of abnormal electrical activity in the brain. About 65 million people worldwide are estimated to have epilepsy [1]. Many epilepsy patients can be treated effectively with currently available antiepileptic drugs (AEDs), but in up to 30% of this patient population adequate seizure control is not achieved [2]. The condition of patients that show drug-resistant seizures can sometimes be improved by non-pharmacological treatment, but even after surgery, 10% of patients continue to experience seizures [2]. For these reasons, the discovery of novel anticonvulsant compounds and the subsequent development of new, alternative AEDs remains an important area of research.

In spite of the fact that the acute pentylenetetrazol (PTZ) rodent seizure model was developed more than 60 years ago, it is still a widely used test for evaluating potential anticonvulsant compounds [3]. In mice, subcutaneous (s.c.) PTZ administration produces a characteristic behavioral pattern of events: ear twitch, vibrissae twitch, straub tail, myoclonic twitch, forelimb clonus, falling and tonic hind limb extension [4]. These seizure behaviors correlate with spiking activity and spike-wave discharges in the cortex as measured by electroencephalography (EEG) [4]. Although the predictive value of the acute PTZ test is high, rodent models have certain disadvantages for large-scale screening applications in comparison with smaller vertebrates, namely: higher cost, lower throughput, the requirement for appreciable amounts of compound, as well as regulatory and ethical considerations.

Zebrafish larvae have recently emerged as a new species for chemoconvulsant-based models of epilepsy [5]–[9]. Zebrafish larvae offer several advantages as an *in vivo* screening platform for drug discovery due to their high genetic and physiologic homology to humans [10]. Per week, a single mating pair of adult zebrafish can generate up to 200 offspring, which develop rapidly *ex utero*. Compounds can be added directly to the surrounding medium and are absorbed by larvae either through the gastrointestinal tract, skin or gills. Due to their small size, zebrafish larvae can live in small volumes, and therefore require only microgram amounts of compound per test. With regard to anticonvulsant screens, medium- to high-throughput screening is possible in 96-well format using an automated locomotor tracking system for the quantification of larval movement.

To track changes in brain activity of a small subject such as the zebrafish larva, local field potential recordings have been the method of choice [5]. Epileptiform activity recorded from zebrafish brains includes interictal and ictal-like discharges and is similar to the neural activity described for hippocampal slice recordings from rodent brains [5], [11]. Interictal-like discharges recorded from zebrafish larval brains have been described as small and frequent, while ictal-like discharges possess high amplitude and occur infrequently [12].

The zebrafish PTZ seizure model has already been partly described: zebrafish larvae exposed to this chemoconvulsant, exhibit increased locomotor activity and seizure-like stereotypic behavior in a concentration-dependent manner [5]. PTZ-treated larvae show epileptiform electrographic discharges on field potential recordings from the optic tectum [5], [8]. Nevertheless, the systematic direct comparison between a decreased locomotor activity in larval zebrafish and true anti-seizure/EEG modulating activity of AEDs has not yet been reported [7]. Here we study the effectiveness of 13 AEDs against PTZ-induced increase in movement and epileptiform electrical activity to assess both the advantages and limitations of this animal model for primary screening in drug discovery.

## Materials and Methods

### Animals

Zebrafish (*Danio rerio*) stocks of the Ekkwill strain (Ekkwill Tropical Fish Farm, Gibonston, Florida) were maintained at 28.5°C, on a 14/10 hour light/dark cycle under standard aquaculture conditions, and fertilized eggs were collected via natural spawning. Embryos were reared under constant light conditions in embryo medium: 1.5 mM HEPES, pH 7.6, 17.4 mM NaCl, 0.21 mM KCl, 0.12 mM MgSO_4_, and 0.18 mM Ca(NO_3_)_2_) in an incubator at 28.5°C. In our experimental setup, we found that larvae displayed the most consistent basal activity levels when raised under constant light conditions. For all measurements described, larvae of 7 days post-fertilization (dpf) were used.

All zebrafish experiments carried out were approved by the Ethics Committee of the University of Leuven (Ethische Commissie van de KU Leuven, approval number P05090) and by the Belgian Federal Department of Public Health, Food Safety & Environment (Federale Overheidsdienst Volksgezondheid, Veiligheid van de Voedselketen en Leefmileu, approval number LA1210199).

### Drugs

The following anticonvulsants: carbamazepine (CBZ), ethosuximide (ETS), oxcarbazepine (OXC), lamotrigine (LTG), levetiracetam (LVT), zonisamide (ZSM), primidone (PMD), topiramate (TPR), and aspirin (ASP; negative control) were purchased from Sigma. Other AEDs used in this study were: diazepam (DZP; Roche), gabapentin (GBP; Fluka), tiagabine (TGB; Chemos), sodium valproate (VPA; Sanofi-Aventis), and phenytoin (PHT; Acros). All compounds were dissolved in DMSO and diluted in embryo medium to achieve a final DMSO concentration of 1% w/v. Embryo medium prepared with DMSO to a final concentration of 1% w/v served as a vehicle control (VHC). Pentylenetetrazol was also purchased from Sigma and was dissolved to 40 mM (2x stock) in embryo medium.

### Toxicological Evaluation

Zebrafish larvae were incubated with AEDs at 28.5°C in complete darkness. After 90 min, each larva was individually checked under the microscope for the following signs of acute locomotor impairment: hypoactivity, decreased or no touch/escape response upon a light touch of the tail with a fine needle [13], [14], loss of posture, body deformation, exophthalmos (bulging of the eyes out of their sockets), slow or absent heartbeat, and death. After an overnight incubation (18 hours, 28.5°C, complete darkness), assessment of larvae for the same above-mentioned signs of toxicity was repeated. A larva was considered normal if it could cover a distance twice its body length. A shorter distance travelled or movement in the same place was scored as a decreased or impaired touch response. No visible movement upon a touch stimulus was counted as no response. The MTC was thus defined as the maximum concentration that did not cause death and where not more than two out of 12 larvae exhibited any sign of locomotor impairment including no touch response after an 18-hour incubation period. A decrease in spontaneous movement with retained ability to swim away in response to touch was considered an acceptable AED concentration for further testing.

### Evaluation of Anticonvulsant Activity

**Figure 1:**
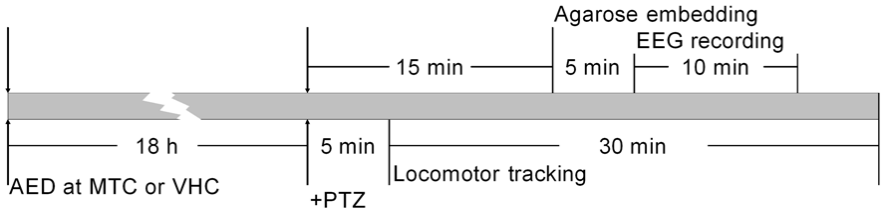
Schematic comparison of the experimental timelines used for the behavioral tracking and EEG assays. Electrographic recordings (EEG) are depicted in the upper portion, while locomotor tracking is depicted in the lower portion. Treatment parameters, order in which they are applied and the key time-points for each experimental protocol are indicated by the arrows.

- **Movement tracking system.** Six-dpf larvae were pre-incubated in 100 µl of AED or VHC for 18 hours in individual wells of a 96-well plate at 28.5°C in the dark (for timeline schematic see Figure 1). Ten to twelve larvae were used per treatment parameter and per experiment. The concentrations of AEDs used are described in the results section. After the pre-incubation, 100 µl of embryo medium or 100 µl of a 40 mM PTZ solution was added to obtain a final concentration of 20 mM [7], [15]. Larvae were allowed to habituate for 5 min in a dark chamber of an automated tracking device (ZebraBox™ apparatus; Viewpoint, Lyon, France). The total locomotor activity was then quantified using ZebraLab™ software (Viewpoint, Lyon, France) [15]. Total movement or activity was expressed in “actinteg” units. The actinteg value of the ZebraLab™ software is defined as the sum of all image pixel changes detected during the time slice defined for the experiment. All tracking experiments were performed at least in triplicate.
- **EEG recordings.** Each larva was pre-incubated in 400 µl of AED or VHC for 18 hours in individual wells of a 24-well plate at 28.5°C in the dark. An equal volume of 40 mM PTZ (2x solution) was then added to each well and the larvae were exposed to the chemoconvulsant for 15 min (Figure 1). A larva was then embedded in 2% low-melting-point agarose, a glass electrode filled with artificial cerebrospinal fluid composed of: 124 mM NaCl, 2 mM KCl, 2 mM MgSO_4_, 2 mM CaCl_2_, 1.25 mM KH_2_PO_4_, 26 mM NaHCO_3_ and 10 mM glucose (resistance 1–5 MΩ), was placed into the optic tectum and recordings were performed in current clamp mode, low-pass filtered at 1 kHz, high-pass filtered 0.1 Hz, digital gain 10, sampling interval 10 µs (MultiClamp 700B amplifier, Digidata 1440A digitizer, both Axon instruments, USA). The recordings started each time exactly 5 min after removal of the larva from proconvulsant solution and were continued for 10 min. Thus, EEG recordings were performed consistently from min 20 through 30 following exposure to PTZ (Figure 1). Recordings from eight or nine larvae were taken per experimental condition. Epileptiform activity was analyzed according to the duration of spiking paroxysms as described previously [15]. Briefly, we compared the total number, the average duration and the cumulative duration of epileptiform discharges in treated larvae. Spikes were categorized as interictal-like (<3 s) or ictal-like (>3 s) [12] with amplitudes exceeding three times the background noise with the aid of Clampfit 10.2 software (Molecular devices corporation, USA).

### Statistical Analysis

Locomotor behavior data was first normalized against the VHC+PTZ controls from the same tracking experiment and the normalized data from replicate tracking runs subsequently pooled together. AED+PTZ treatment groups were compared to VHC+PTZ groups and VHC+medium groups using repeated measures (mixed model) ANOVA. Both electrographic parameters and the average total movement within 30 minutes were compared using one-way ANOVA followed by Dunnett’s multiple comparison test (GraphPad Prism software).

## Results

### Toxicological Evaluation

The maximum-tolerated concentrations (MTCs) of drugs determined and used for further experiments are presented in Table 1. Notably however, larvae treated with 300 µM LTG alone were normal but displayed body distortions and became paralyzed or died after addition of PTZ. Therefore, to avoid toxicity as a result of the combination of these two compounds the experiment was repeated with 100 µM LTG and this same concentration was used for tectal field recordings. Vehicle-treated fish mostly escaped before being touched, had normal heart rates, and displayed no other signs of toxicity.

**Table 1:** Comparison of AED activity in the zebrafish and rodent acute PTZ models.

AED	Zebrafish locomotor assay			Zebrafish EEG	Rodent behavioral assay	Rodent EEG
	MTC	Integration interval				
		30 min	5 min			
**Carbamazepine**	100 µM	**–**	**–**	**–**	**–** [^3^], [4], [16], [17], [19], [27]	**–** [^4^]
**Diazepam**	16 µM	**–**	**+**	**+**	**+** [^4]^, [19]	**+** [^4^]
**Ethosuximide**	10 mM	**–**	**+/−**	**+**	**+** [^4]^, [16], [19]	**+** [^4]^, [18]
**Gabapentin**	25 mM	**–**	**–**	**–**	**+** [4], [16], **+/−** [^3^]	**+** [^4^]
**Lamotrigine**	100 µM	**+**	**–**	**–**	**–** [^16]^, [19], [21]	**–** [^28^]
**Levetiracetam**	10 mM	**–**	**+/−**	**–** ^a^	**–** [^16]^, [19], [21], [29]–[32]	**+** ^SWD ^ [^20^]
**Oxcarbazepine**	250 µM	**–**	**+/−**	**–**	**+** [^17]^, [19], [33], **–** [^3]^, [16]	NA
**Phenytoin**	100 µM	**–**	**–**	**–**	**–** [^4]^, [16], [17], [19], [27]	**+/−** [^4^], **–** [^27^]
**Primidone**	750 µM	**–**	**–**	**–**	**+** [^21^]	NA
**Tiagabine**	100 µM	**–**	**–**	**+**	**+** [^16]^, [19], [33]	NA
**Topiramate**	200 µM	**+**	**+**	**–**	**+/−** [^27^], **–** [^4]^, [16], [19], [21]	**–** [^4^]
**Valproate**	1 mM	**+**	**+**	**+**	**+** [^4]^, [16], **–** [^19^]	**+** [^4^]
**Zonisamide**	300 µM	**–**	**+**	**–**	**+** [^16]^, [19], **–** [^17^]	NA
**Aspirin**	50 µM	**–**	**–**	**–**		

An AED is indicated as positive for 30-min integration intervals if it significantly (p<0.05) decreased locomotor activity. We considered an AED positive (+) in the 5-min time slices if there were one or more points significantly different from the PTZ-treated group (p<0.01 or better). Slight activity (+/−) was indicated when only one time point was significantly lower (p<0.05) than the corresponding control point. ^a^, spike-wave discharges (SWD) can not be directly measured in zebrafish tectal field recordings; NA, data not available.

### Proconvulsant Treatment

**Figure 2:**
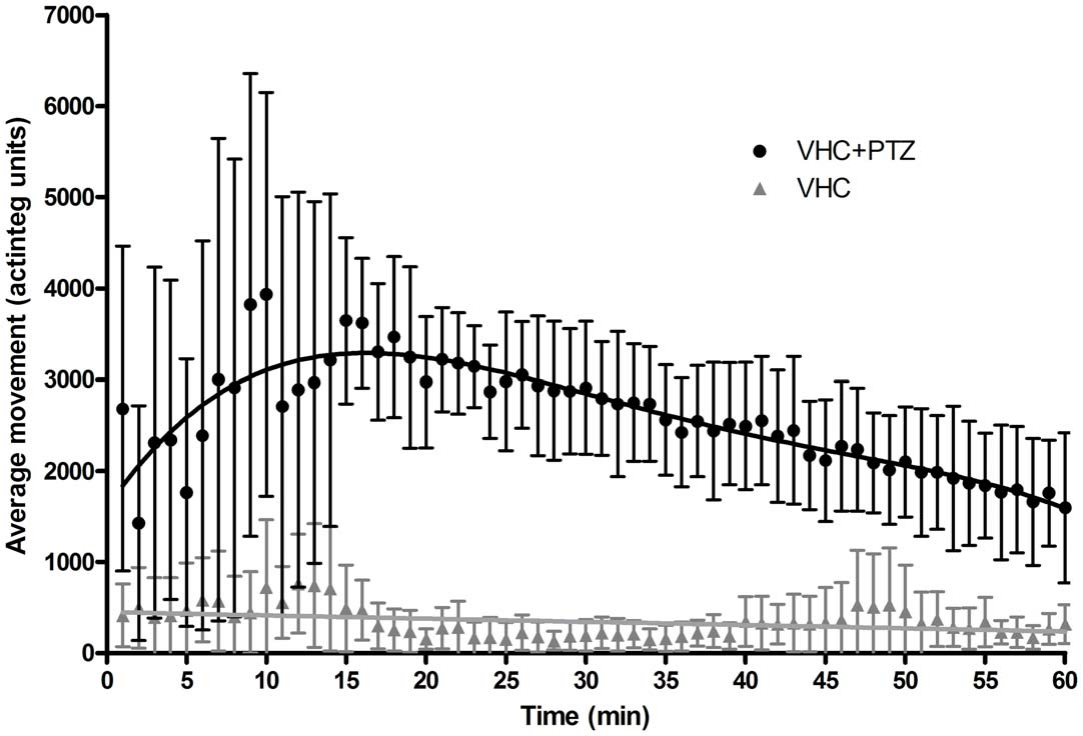
Behavioral profile of zebrafish larvae during a one-hour exposure to vehicle or PTZ. The average movement (y-axis) of vehicle- and 20 mM PTZ-treated larvae are denoted by closed triangles (grey) and closed circles (black) respectively. The mean movement ± SD of 12 larvae is depicted per minute (x-axis) of the tracking session. By 15 min, the frequency of convulsion-like episodes in all larvae reached a rate of one or more per minute, which is reflected in the decrease in SD after this time point.

**Figure 3:**
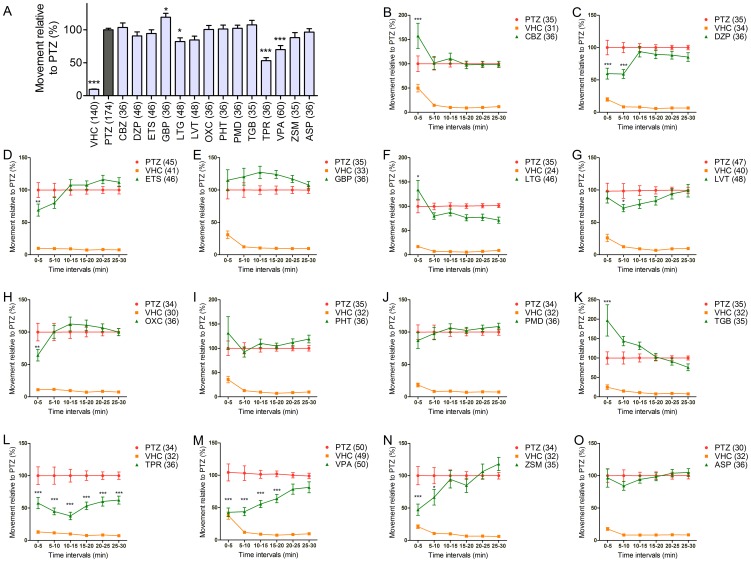
Behavioral profile of zebrafish larvae to AEDs. (A) The top graph depicts the average larval locomotor activity within 30 minutes (y-axis) relative to VHC+PTZ (PTZ) control, as depicted by the average % ± SEM; treatment groups where average movement was significantly decreased compared to PTZ (one-way ANOVA) are marked *, **, and *** (p<0.05, p<0.01, and p<0.001, respectively). (B-O) The average total movement (y-axis) of larvae treated with VHC only or PTZ with either VHC or AEDs. The average larval movement is depicted per 5 min interval (x-axis) of the tracking session. Time points when the average movement was significantly decreased compared to PTZ control (repeated measures ANOVA) are marked *, **, and *** (p<0.05, p<0.01, and p<0.001, respectively); standard errors are shown. F values (treatment group-dependent variability): (A) 55.88, (B) 77.26, (C) 144.3, (D) 107.6, (E) 124.7, (F) 65.73, (G) 80.58, (H) 79.56, (I) 70.39, (J) 153.2, (K) 70.29, (L) 80.36, (M) 97.76, (N) 68.3, (O) 137.8. Total number of larvae used are indicated in brackets.

Zebrafish larvae exposed to PTZ exhibited signs of agitation within seconds of contact with the proconvulsant. The larvae swam along the periphery of the well (thigmotaxis), thus displaying Stage I seizure-like behavior as described previously [5]. This was succeeded by rapid 'whirlpool'-like movement, and then followed by a short pause before swimming in a rapid, jerky manner with occasional body-stiffening and loss of posture (larva turning onto its side or back). These events can be likened to tonic and clonic seizure phases in mammals (Stages II-III) [7]. The working concentration was defined as a concentration that induced a significant increase in locomotor activity for the majority of larvae tested within 30 min. We selected the concentration for PTZ at 20 mM for the behavioral test, which is in line with the effective concentrations (15 mM, 20 mM) reported previously [5], [7], [9], [15]. Movement increase upon PTZ exposure peaked at around 15 min after the start of the experiment (Figure 2), and then continued for at least another hour. Notably a decline in total larval movement between 20 and 60 min was observed and is most likely due to the majority of larvae spending more time in seizure stage III, resulting in loss of posture and a consequent decrease in locomotor activity. In addition, initial pilot experiments revealed that in the first 15 min immediately after addition of PTZ, larval activity levels are highly erratic, but become more uniform thereafter, as the larvae start to undergo convulsions every minute or even more frequently (Figure 2). Moreover, the addition of PTZ solution (or vehicle) to the larvae and the transfer of the 96-well plate to the tracking chamber acted as external stimuli and caused larvae to initially become more active. Hence, for all subsequent experiments, we allowed larvae to ‘habituate’ in the dark (except for the infrared illumination required by the camera to capture larval movements) for 5 min prior to the start of a tracking session. Although, the level of larval activity is much more uniform 15 min after addition of PTZ, we chose to commence tracking immediately after the 5 min habituation period because pilot experiments also revealed that some AEDs were capable of significantly reducing larval locomotor activity already within the first 5 min after the start of the tracking session (Figure 3). Finally, tracking for 30 min was chosen for convenience, because longer exposure to PTZ did not increase the level of locomotor activity compared to controls further (signal to background ratios for 30 min and 1 hour periods were 9.4 and 8.5 respectively).

### Test of AEDs in the Larval Zebrafish PTZ Assay

Out of a panel of thirteen commercially available AEDs tested against PTZ, TPR (p<0.001), VPA (p<0.001), and LTG (p<0.05) decreased PTZ-induced movement compared to VHC+PTZ controls within the 30-min integration time, while GBP increased this parameter (p<0.05). All other AEDs were not active at their MTCs (Figure 3, Table 1).

However, a more detailed analysis of the 30-min tracking period into 5-min intervals revealed the kinetics of the larval response to PTZ more precisely (Figure 3). The rationale behind this type of analysis was to detect transient effects or increased latency periods for any of the tested AEDs, taking into consideration the fact that the larvae are subjected to a continuous bath of PTZ throughout the tracking assay (see Discussion). We considered an AED positive if there were more than one point that showed a statistically significant decline in total movement compared to the VHC+PTZ treated group (Table 1, Figure 3). If there was only one time point significantly (p<0.05) lower than the corresponding control, the treatment was marked as slightly active (+/−). Using this analysis method, DZP, TPR, VPA, and ZSM were considered active in decreasing total larval movement while ETS, LVT, and OXC were scored as slightly active (Table 1, Figure 3). With regard to LTG, we observed a statistically significant increase in larval locomotor activity within the first 5-min interval. However, all other time-points showed no significant difference when compared to VHC+PTZ-treated controls. Thus, using the 5-min interval analysis method, LTG was scored as negative.

### Tectal Field Recordings

**Figure 4:**
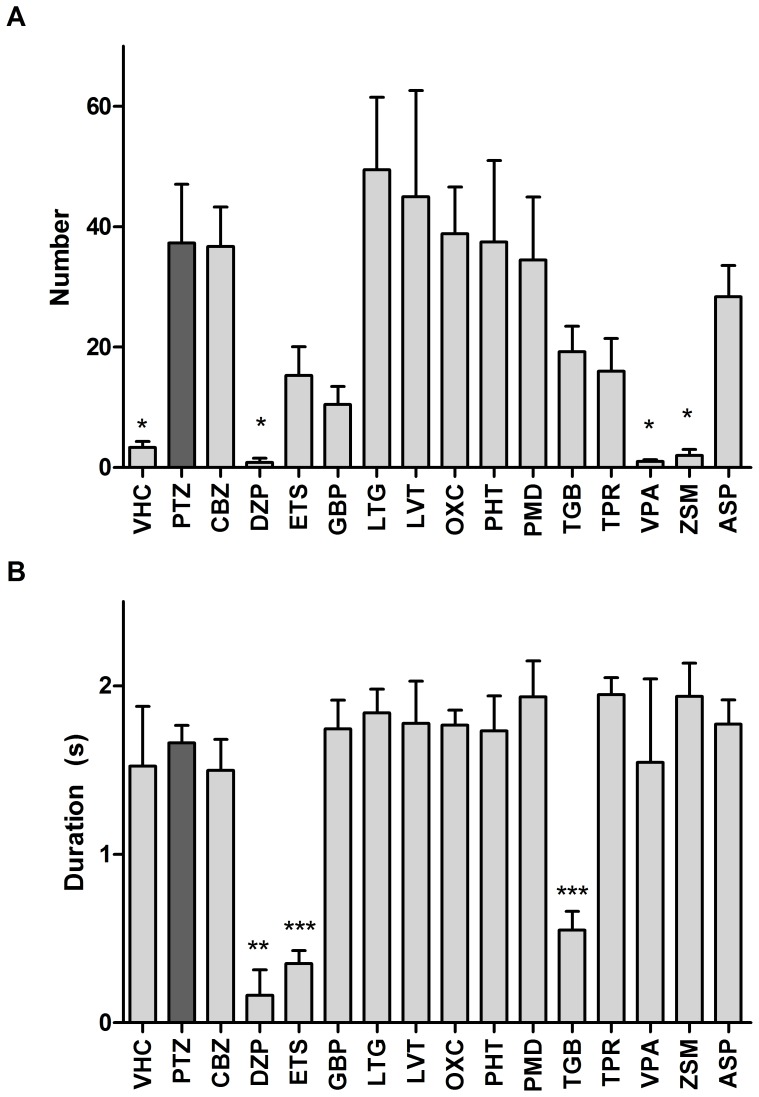
Quantitative analysis of interictal-like electrographic activity in response to AEDs. (A) Total number of interictal-like events within a 10-min recording (mean±SEM); (B) duration of interictal-like events in seconds within a 10-min recording (mean±SEM). Values that were significantly different from PTZ control were determined using one-way ANOVA with *, ** and *** denoting p<0.05, p<0.01 and p<0.001 respectively. The number of recordings analysed were: VHC (n = 8), PTZ (n = 9), CBZ (n = 8), DZP (n = 8), ETS (n = 9), GBP (n = 8), LTG (n = 8), LVT (n = 8), OXC (n = 8), PHT (n = 8), PMD (n = 8), TGB (n = 8), TPR (n = 8), VPA (n = 8), ZSM (n = 8), ASP (n = 8); F values (treatment group-dependent variability): (A) 4.298, (B) 6.602.

The analysis of interictal-like electrographic activity after AED exposure revealed that DZP, VPA and ZSM significantly decreased the total number of interictal-like spikes. In addition, the average duration of an interictal event was significantly shortened by ETS, DZP and TGB (Figure 4).

**Figure 5:**
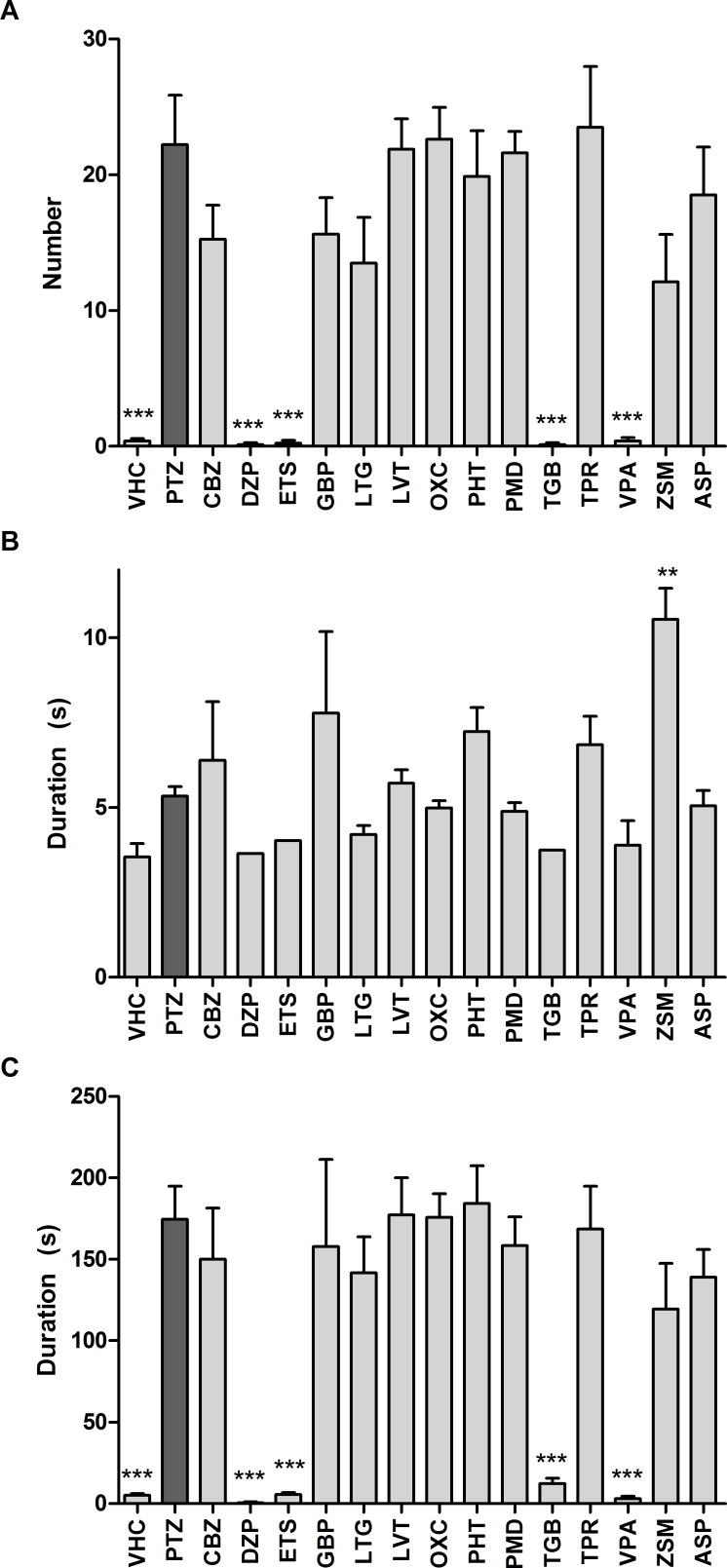
Quantitative analysis of ictal-like electrographic activity in response to AEDs. (A) Number of ictal-like events within a 10-min recording (mean±SEM); (B) duration of ictal-like events within a 10-min recording (mean±SEM); (C) total cumulative duration of all types of epileptiform activity measured. Values that were significantly different from PTZ control were determined using the one-way ANOVA with *, ** and *** denoting p<0.05, p<0.01 and p<0.001 respectively. The number of recordings analysed were: VHC (n = 8), PTZ (n = 9), CBZ (n = 8), DZP (n = 8), ETS (n = 9), GBP (n = 8), LTG (n = 8), LVT (n = 8), OXC (n = 8), PHT (n = 8), PMD (n = 8), TGB (n = 8), TPR (n = 8), VPA (n = 8), ZSM (n = 8), ASP (n = 8). F values (treatment group-dependent variability): (A) 13.45, (B) 3.056, (C) 11.41.

**Figure 6:**
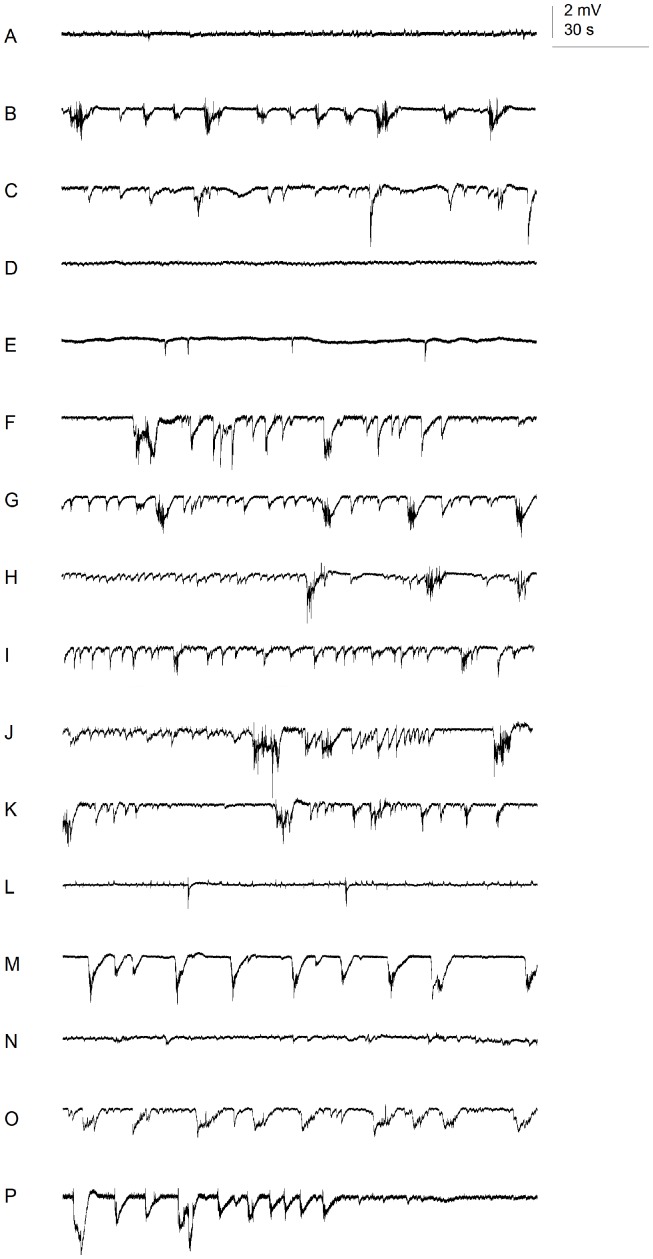
Electrographic activity in zebrafish optic tecta: fragments of representative recordings. (A) Vehicle only; (B – P) 20 mM PTZ with the following pre-treatment: (B) VHC; (C) CBZ; (D) DZP; (E) ETS; (F) GBP; (G) LTG; (H) LVT; (I) OXC; (J) PHT; (K) PMD; (L) TGB; (M) TPR; (N) VPA; (O) ZSM; (P) ASP. Recordings were performed in current clamp mode, low-pass filtered at 1 kHz, high-pass filtered 0.1 Hz, digital gain 10, sampling interval 10 µs.

DZP, ETS, TGB, and VPA dramatically decreased the number of ictal discharges, as well as the total cumulative duration of all types of epileptiform activity (Figure 5, Table 1). The average duration of ictal spikes was significantly increased after exposure to ZSM (Figure 5B). Other AEDs did not significantly change any of the ictal discharge parameters analyzed (see Figure 6 for representative time fragments of recordings of control and treated larvae).

## Discussion

In this study, we demonstrate that the larval zebrafish behavioral response to PTZ, either alone or in combination with AEDs, is largely indicative of changes in electrographic activity in the brain. By aligning our findings in zebrafish with previously reported rodent data on the effects of various AEDs in the acute PTZ test, we also found a good correlation between zebrafish and rodent data in both the behavioral and EEG assays.

DZP, ETS, VPA and TGB counteracted PTZ-evoked electrographic seizures in both rodents [4], [16]–[18] and zebrafish (our data), while CBZ, LTG, PHT, and TPR were inactive in both species [4,15,16,18,19, and our data]. DZP, ETS, and VPA also inhibited convulsions in the locomotor tracking assay, while TGB showed no activity. Thus, TGB turned out to be a false negative in the zebrafish behavioral assay. The reason for this remains unclear. One possibility is that TGB exerts additional peripheral or off-target effects independent of its ability to suppress PTZ-induced seizures. In contrast, LVT, TPR, OXC, and ZSM inhibited seizure-like behavior in zebrafish larvae, but failed to inhibit total seizure duration and/or frequency in the EEG recordings. Notably, ZSM even worsened electrographic seizures. We postulate that the observed decrease in larval locomotor activity is due to a prolonged period wherein larvae remain in seizure stage 3 [5]. In this seizure stage, larvae spend a good portion of their time on their side or ‘belly up’ due to a loss of balance. The end result is a general decrease in large movements. A similar phenomenon is observed in rodents undergoing status epilepticus where hardly any movement is observed, if at all. With regard to LVT, it also failed to alter all EEG parameters in rodent seizure assays, except for a slight reduction in spike wave discharges [20], a parameter not measured in our study. Nevertheless, our zebrafish EEG data correlate well with those reported for mice by Watanabe et al. [4].

The results for LTG were rather ambiguous for the locomotor assay. LTG exerted a mild but statistically significant (p<0.05) activity in lowering PTZ-induced movement in the 30-minute interval analysis, but came up consistently negative in the 5-min interval analysis. Given that LTG was only ‘borderline’ positive in the 30-min interval analysis, negative for the 5-min interval analysis and negative in the EEG assay, then the overall conclusion is that LTG is not active in the larval PTZ seizure assay, a result that is perfectly in line with published rodent data (see Table 1).

PMD and GBP did not inhibit PTZ-induced locomotor activity in zebrafish larvae but were reported to be effective in the equivalent rodent assays [4], [21]. PMD is metabolized to phenobarbital and phenylethylmalonamide – both potent AEDs in mammals [22]. A previous report by Baraban and colleagues showed that phenobarbital was only mildly effective in suppressing PTZ-evoked seizures in zebrafish [5]. PMD's biotransformation rate and pharmacokinetic profile in zebrafish larvae remains to be determined – a general issue for all AEDs tested in zebrafish.

Although GBP contains a GABA moiety in its chemical structure, it is instead thought to exert its antiepileptic activity through the interaction with α2-δ subunits of voltage-gated Ca^2+^ channels [22]. Voltage-gated Ca^2+^ channels have been cloned and characterized in developing zebrafish [23], [24], and are already expressed and active within the first several days of development. Thus, the fact that GBP was inactive in our assays may simply be due to its hydrophilic properties as compounds with negative logP values tend to be less active and/or poorly taken up in zebrafish larvae [25,26, our own unpublished observations].

By administering AEDs at concentrations that on their own did not cause locomotor impairment in zebrafish larvae, the total number of AEDs shown to be active in the acute PTZ assay was appreciably lower in comparison with the results of the previous report by Berghmans and colleagues [7]. Admittedly, we used very stringent criteria in the choice of AED concentrations, and this difference most likely accounts for some of the discrepancies observed. However, even in cases where the AED concentration used was the same, we still observed differences in activity. Furthermore, differences in the ability of several AEDs to induce hypoactivity were also observed [7]. Although we followed very similar protocols (e.g. overnight pre-treatment with AED before addition of PTZ, same concentration of PTZ used, same assay temperature 28.5°C applied, and AED pre-incubation, habituation period and tracking assay performed in complete darkness), additional factors that may have influenced the outcome of our study are: 1) strain differences (WIK, Berghmans; Ekkwill, our study), 2) tracking software (Noldus, The Netherlands, Berghmans; Viewpoint, France, our study), and 3) total tracking time (60 min, Berghmans; 30 min, our study). With regard to strain differences, pilot tests carried out while designing our experimental protocol revealed that strain differences (Ekkwill vs. AB strain) and tracking periods did not result in any significant changes in experimental outcome.

On the other hand, we found a good correlation between our EEG data and those reported by Baraban and colleagues [5], [25], [26], despite differences in protocol, namely: 1) PTZ concentration (15 mM PTZ, Baraban; 20 mM PTZ, our study), 2) Strains used (TL, Baraban; Ekkwill, our study), 3) Timing of drug application (PTZ added before AED, Baraban; AED added before PTZ, our study), and 4) Type of EEG parameter/s evaluated (spike amplitude, Baraban; number and duration of epileptiform discharges, our study). The single difference we observed was in the case of ETS. This AED was reported by Baraban and colleagues to be ineffective in decreasing spike amplitude, whereas in our study we found it to significantly reduce both the total number and duration of ictal-like discharges. Nevertheless, a closer look at the EEG recordings we generated from ETS-treated larvae show that spike amplitudes are also not reduced (Figure 6B, 6E and data not shown). Despite major differences in methodology, the results of our locomotor assays are still largely in line with the EEG data generated in our study, with those reported by Baraban and colleagues, and reported rodent data.

Our findings indicate that the locomotor assay has more of a tendency to pick up false positives rather than false negatives. However, a much larger number of AEDs would have to be tested in order to determine the true rate of false positives and false negatives in this assay. Furthermore, the way in which total larval movement is analyzed (i.e. 30-min versus 5-min tracking intervals, can greatly influence experimental outcome, as transient or mild activity of a drug or compound may be masked by combining total activity over longer time periods. It must be emphasized that the locomotor assay is useful as an initial *indicator* of potential anticonvulsant activity, but that secondary assays such as EEG recordings are warranted and would filter out false positives. The development of improved software, in conjunction with faster cameras for automated video tracking analysis, will enable the recognition of more subtle seizure-specific behaviors (e.g. tremor and loss-of-posture events), and improve the accuracy of seizure recognition further. In addition, future development and application of methods that address the pharmacokinetics of small molecules in zebrafish larvae will be of value in the comparison of results from different laboratories.

In summary, despite multiple differences – species, immature vs. mature brain, uptake and metabolism - a good correlation between zebrafish and rodent data was observed, underscoring the biomedical relevance of the zebrafish PTZ seizure model. Similar studies with other zebrafish seizure models will further validate the predictive value of zebrafish for the *in vivo* discovery of novel AEDs.
